# Costal Chondroid Tumors Mimicking Intraabdominal Masses: The Pivotal Role of Computed Tomography in Diagnosis

**DOI:** 10.5334/jbsr.3425

**Published:** 2024-02-20

**Authors:** Gary Amseian, Aleix Jareño, Xavier Tomas

**Affiliations:** 1 Radiology Department, Hospital Clínic de Barcelona, Barcelona, Spain; 2 Radiology Department, Hospital Clínic de Barcelona, Barcelona, Spain; 3 Radiology Department, Hospital Clínic de Barcelona, Barcelona, Spain

**Keywords:** Musculoskeletal, Chondroid, Enchondroma, Chondrosarcoma, Computed Tomography, Rib, Mimick

## Abstract

*Teaching Point:* Costal chondroid tumors can mimic abdominal masses and, when located in the right hypochondrium, may suggest hepatic origin. Computed tomography is essential to determine their origin and nature and to guide appropriate treatment.

## Report of Three Cases

Three cases of patients are presented with masses in the right hypochondrium with likely abdominal origin. In two cases, a computed tomography (CT)-guided percutaneous needle biopsy was performed.

**Figure 1:**
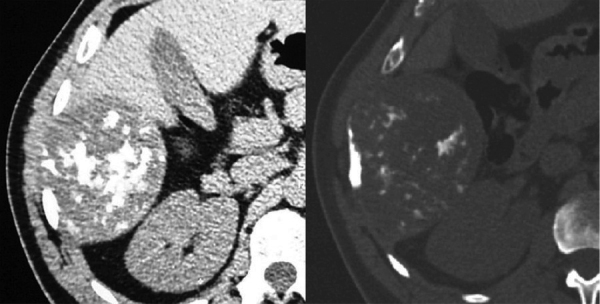
Axial CT scan of a patient with a suspected abdominal mass showing a mass originating in the thoracic wall. Pathological examination demonstrated it to be an enchondroma of the 10th rib.

A 50-year-old man with a history of trauma in the right hemithorax 9 years earlier presented with an abdominal mass. CT (Figure 1) revealed a 9-cm bilobated mass with scattered calcifications originating from the thoracic wall. Histology of the surgical specimen revealed an enchondroma of the 10th rib.

**Figure 2:**
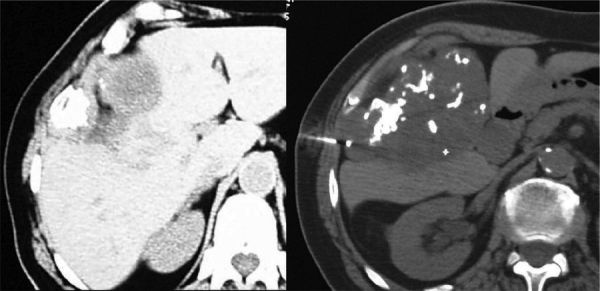
Axial CT scan reveals a mass with coarse calcifications originating from the chest wall. A CT-guided biopsy identified the mass as a chondral tumor.

A 71-year-old woman with a history of type II diabetes and elevated liver enzymes presented for an abdominal ultrasound. A “hepatic mass” was reported, and the CT scan (Figure 2) showed a mass of 8 cm in diameter with coarse calcifications originating from the chest wall. A CT-guided biopsy identified a chondral tumor without cellular atypia, and surgical excision confirmed a low-grade chondrosarcoma of the 9th rib.

**Figure 3:**
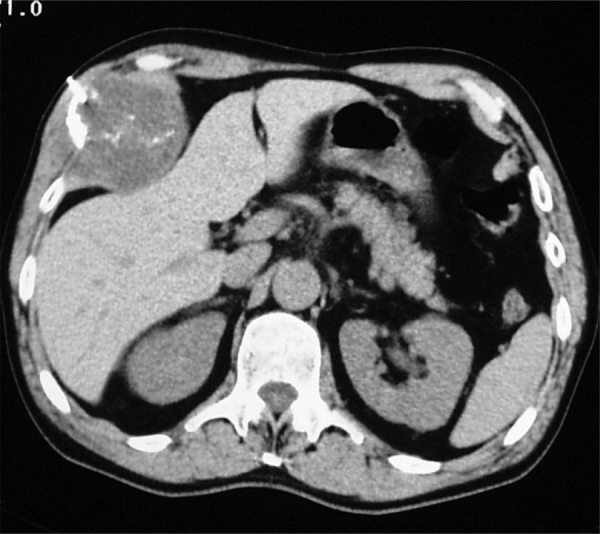
A CT-guided biopsy of a mass with cystic areas and calcifications originating from the 8th rib revealed a malignant chondral tumor.

A 57-year-old man presented with a mass in the right upper abdominal quadrant (Figure 3). A CT scan revealed a 7-cm mass with cystic areas and calcifications originating from the 8th rib. A CT-guided biopsy revealed a malignant chondral tumor; surgical excision confirmed a low-grade chondrosarcoma.

## Comment

Enchondroma (Figure 1) is the second most common benign tumor of the rib after fibrous dysplasia, most commonly located at the costochondral or costovertebral junctions and usually occurring between the 3rd and 5th decades. Typical CT findings include a hypoattenuation lesion with well-demarcated lobulated margins and an internal calcified rings-and-arcs matrix.

Chondrosarcoma (Figures 2 and 3) is the most common primary malignancy of the rib, typically presenting in the 4th to 5th decades, likewise originating at the costochondral or costovertebral junctions. The typical CT appearance includes a soft tissue mass larger than 4 cm, a calcified rings-and-arcs matrix, cortical disruption, and deep endosteal scalloping [1].

Costal chondroid tumors can be mistaken as abdominal masses, especially when originating in the right hypochondrium. CT is essential to make a presumptive diagnosis of its chondroid nature, to assess the extent and to guide a biopsy. There is an overlap in the radiological and cytological appearances of benign and malignant lesions. Radical surgery is the recommended treatment.
